# Pleural Approach to Aberrant Right Subclavian Artery in Aortic Surgery

**DOI:** 10.3400/avd.cr.21-00028

**Published:** 2021-09-25

**Authors:** Kazuhiro Kurisu, Ken-ichi Imasaka, Akira Hashino, Yasutaka Ueno, Akira Shiose

**Affiliations:** 1 Department of Cardiovascular Surgery, Shimonoseki City Hospital, Shimonoseki, Yamaguchi, Japan; 2 Department of Cardiovascular Surgery, Kyushu University, Fukuoka, Fukuoka, Japan

**Keywords:** aberrant right subclavian artery, median sternotomy, thoracic aortic aneurysm

## Abstract

An aberrant right subclavian artery usually arises from the aortic arch just distal to the left subclavian artery and crosses behind the esophagus on its way to the right axillary artery. Several reconstructive techniques of this artery in aortic surgery have been reported but mostly resulted in troublesome procedures. Here, we describe an alternative strategy presenting the occlusion of the aberrant right subclavian artery through the right pleural approach followed via extraanatomical axillary artery bypass. This surgical approach might be a simple and safe option for the aberrant right subclavian artery.

## Introduction

An aberrant right subclavian artery (ARSA) is a rare anomaly and is usually located behind the esophagus and main bronchus.^1)^ Several reports describe its treatment in aortic surgery,^2–6)^ but procedures in the posterior mediastinum are often necessary.^2,3)^ We introduced an extraanatomical axillary artery bypass following the occlusion of the ARSA just at the right side of the thoracic vertebra via the right pleural approach in median sternotomy.

## Case Report

**Figure 1:**
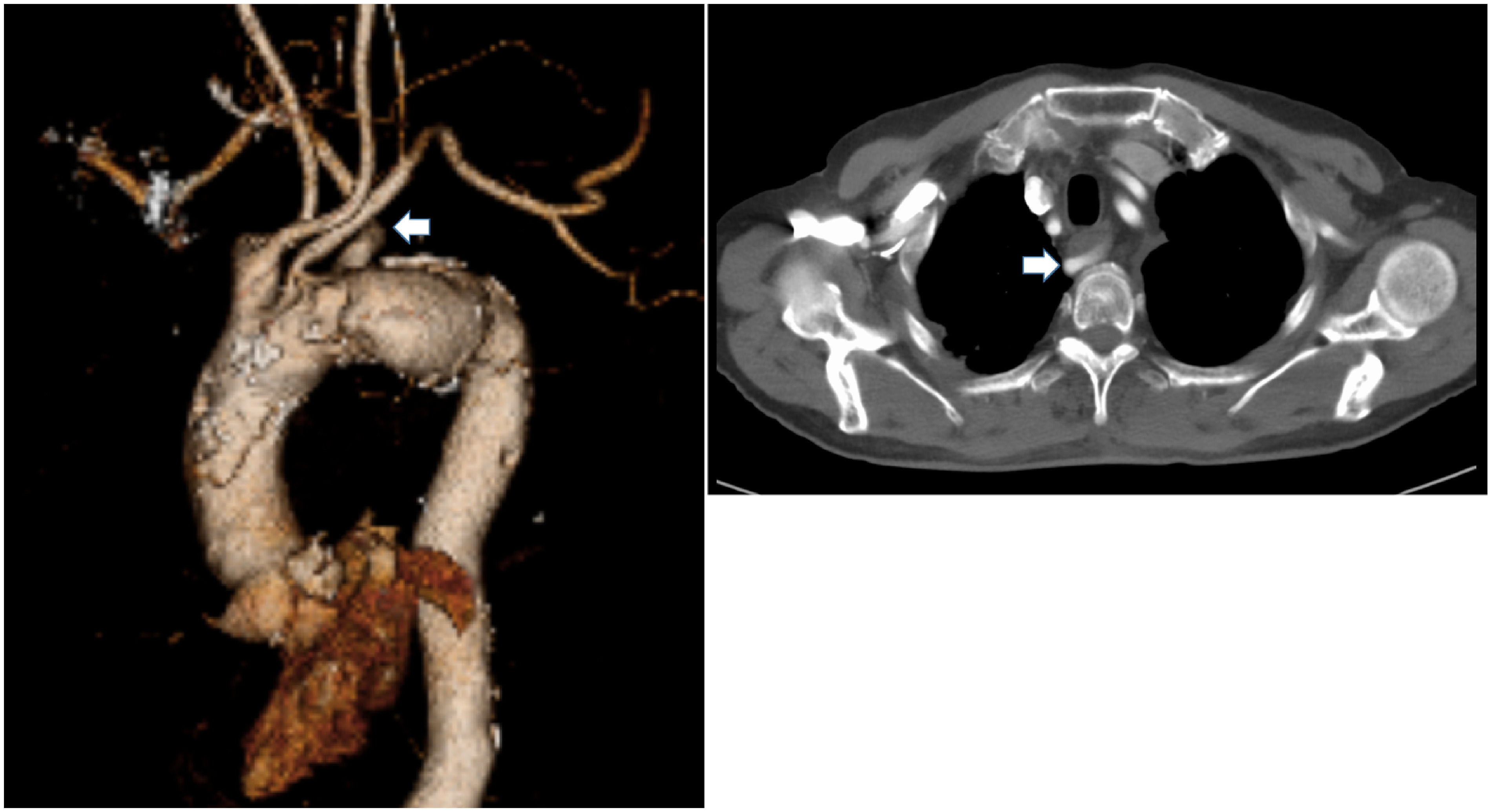
Fig. 1 Preoperative computed tomographic angiography in the left anterior oblique view presented a fusiform-type aneurysm in the distal aortic arch, a common carotid trunk, and an aberrant right subclavian artery (arrow) originating from the back of the aortic arch (left). The axial view showed an aberrant right subclavian artery passing behind the esophagus and trachea (right).

An 82-year-old man incidentally presented with a protruded shadow adjacent to the aortic knob in chest radiography during an assessment of the renal dysfunction. Computed tomographic scanning revealed a fusiform-type aneurysm with a diameter of 56 mm in the distal aortic arch. Aneurysmal change extended to the aortic arch around the origin of ARSA and the left subclavian artery. The patient was found to also have a common carotid trunk and an ARSA independently originating from the back of the aortic arch distal to the left subclavian artery. This artery passed behind the esophagus and trachea and connected the axillary artery through the right side of the thoracic vertebra (**Fig. 1**). Bilateral vertebral arteries ordinarily arose from the superior aspect of the central subclavian arteries. We applied the open repair but not the endovascular repair and decided to perform total arch replacement in combination with frozen elephant trunk and reconstruction of bilateral axillary arteries via the extraanatomical bypass. The occlusion of proximal ARSA was planned just at the right side of the thoracic vertebra in the right pleural approach to avoid troublesome procedures in the deep mediastinum.

**Figure 2:**
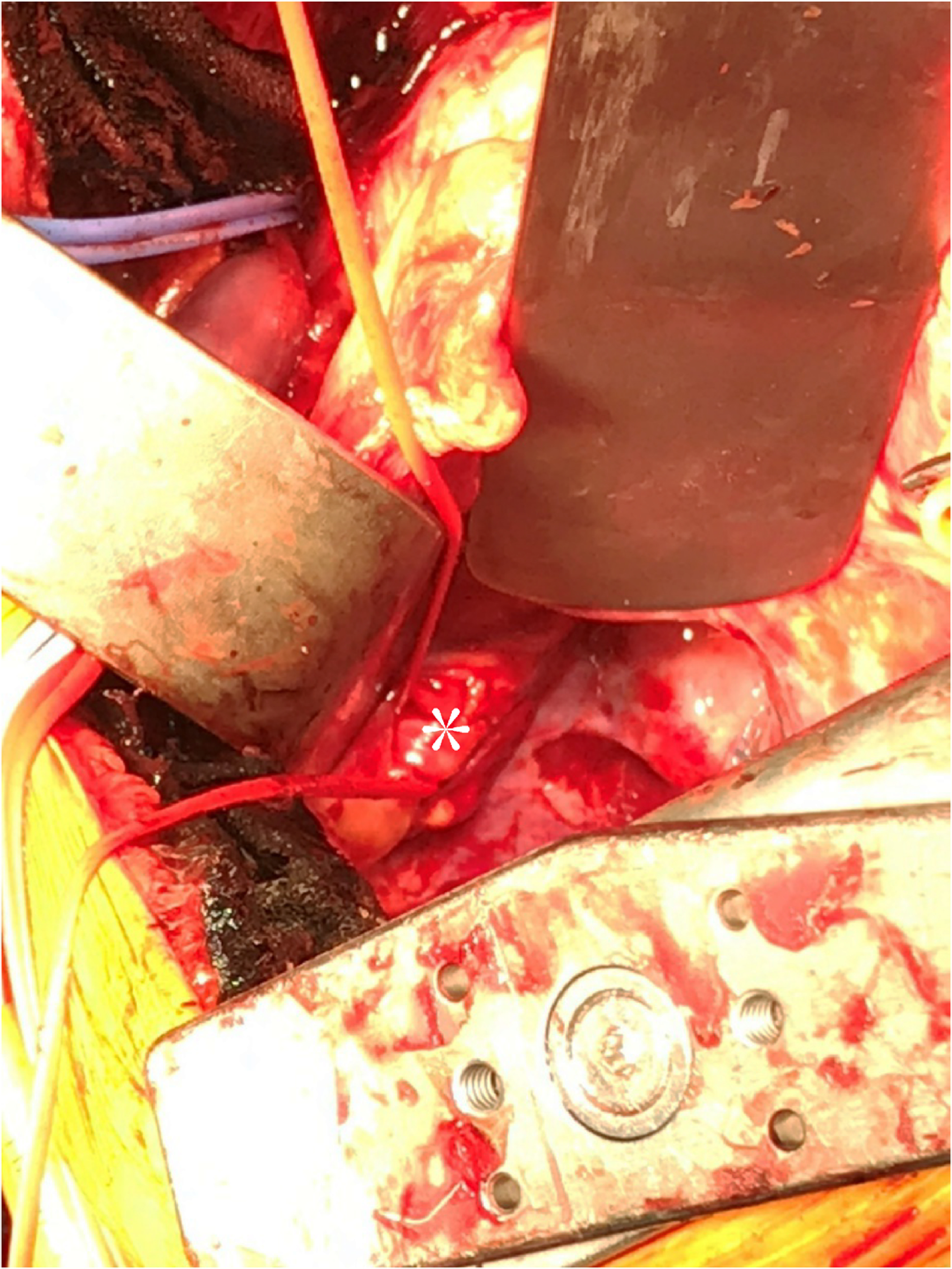
Fig. 2 Operative image displayed an aberrant right subclavian artery (asterisk) through the pleural approach.

Simultaneous preparation for bilateral axillary artery accesses was performed, and a standard median sternotomy was then made. Two pieces of 9 mm vascular grafts (J graft, Lifeline, Tokyo, Japan) were sutured to the bilateral axillary arteries respectively in an end-to-side fashion, and cardiopulmonary bypass was initiated with perfusion via these arteries and atrial drainage with a two-staged cannula. The right pleura was opened during the temporary cessation of mechanical ventilation. The ARSA was easily identified just at the right side of the thoracic vertebra and encircled with a vascular elastic band following the division of the parietal pleura (**Fig. 2**). After systemic cooling, the systemic circulatory arrest followed by aortic opening was then performed with a rectal temperature of 22.9°C. Antegrade selective cerebral perfusion was started through vascular grafts connected to the bilateral axillary arteries and perfusion catheters directly cannulated into each right and left common carotid artery.^7,8)^ The ARSA was occluded using a surgical clip (Hem-o-lok, Teleflex, Tokyo, Japan) along the previously placed elastic band through the pleural approach. The left subclavian artery was ligated at the beginning. The aorta was transected between the common carotid trunk and the left subclavian artery, and a 29 mm stented graft (Frozenix, Lifeline, Tokyo, Japan) was inserted. Its stump was anastomosed to a 26 mm four-branched graft (J graft, Lifeline, Tokyo, Japan). The systemic circulation was resumed with a 9 mm side branch of the four-branched graft, and the common carotid trunk was anastomosed to the first 11 mm side branch. After the grafts connected to the axillary arteries were passed into the mediastinal space, bilateral axillary arteries were reconstructed via the extraanatomical bypass using second and third 9 mm side branches of the arch graft. The cardiopulmonary bypass was terminated after sufficient rewarming.

**Figure 3:**
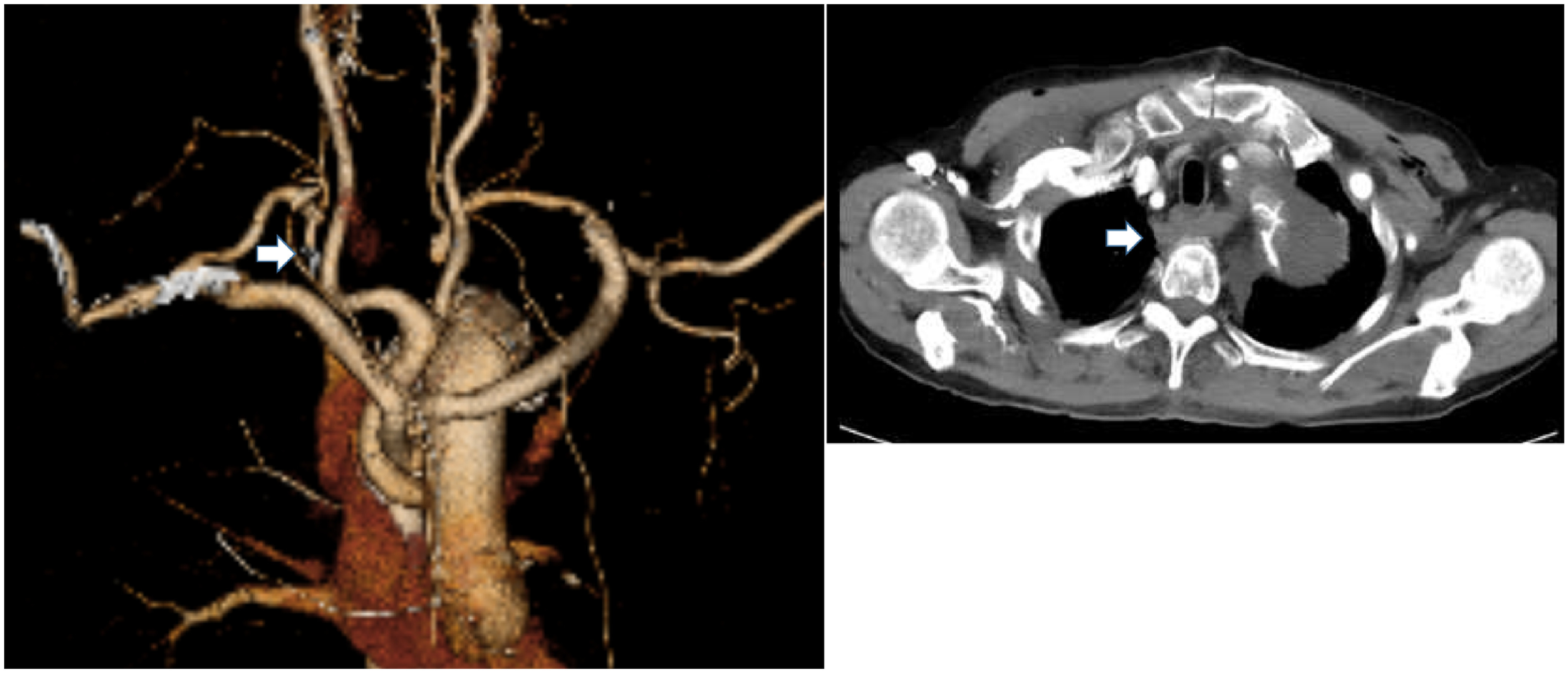
Fig. 3 Postoperative computed tomographic angiography in the frontal view presented a successful bloodstream to the bilateral carotid and subclavian arteries. Note that an aberrant right subclavian artery (arrow) was occluded by using the surgical clip (left). The axial view showed proximal aberrant right subclavian artery no longer contrasted (right).

The mechanical ventilator support was 16 h, and thereafter, the patient experienced an uneventful postoperative course. Postoperative computed tomographic findings showed good reconstruction of the aortic arch and successful bloodstream to the bilateral carotid and subclavian arteries (**Fig. 3**).

## Discussion

The ARSA is a rare aortic arch anomaly, occurring in 0.4% to 2.0%.^1)^ Most of the patients are asymptomatic, but it can lead to serious vascular complications including aneurysmal dilatation, aortic dissection, and compression of the trachea and esophagus.^1)^

The surgical approach to the ARSA is very hesitating in aortic arch surgery performed through the median sternotomy. In previous reports, the procedures associated with the treatment of the ARSA are needed to operate in the deep mediastinum adjacent to the trachea and esophagus or additional approaches through a supraclavicular or lateral thoracotomy approach.^2,3)^ These procedures are very troublesome and time consuming. Although not frequently, serious complications including recurrent nerve paralysis and esophageal dysphagia were reported.^5)^ Moreover, some cases necessitated plugging of the proximal portion of ARSA using the endovascular techniques.^5)^ Therefore, we adopted an alternative approach for the ARSA just at the right side of the thoracic vertebra through the right pleural approach in the median sternotomy. This strategy to treat the ARSA can be performed very easily and promptly. A series of these procedures was exactly completed in several minutes. We expect that our strategy is beneficial, but further investigations are necessary because there is a potential for complications related to the graft anastomosis to axillary arteries.

The extraanatomical axillary artery bypass following the graft anastomosis to the axially artery is a unique strategy.^5–8)^ These axillary artery grafts were initially used for the systemic perfusion followed by cerebral perfusion and later for the reconstruction of a bypass route to the axillary artery.^7,8)^ This strategy should also lead to the simplicity and safety of the surgery because the procedures in the deep mediastinum are avoided.^5,6)^

## Conclusion

We describe the technique presenting the occlusion of ARSA through the right pleural approach followed by the extraanatomical axillary artery bypass. Although the indication is limited, this approach might be an alternative option for the treatment of ARSA.
